# Molecular snapshots of APE1 proofreading mismatches and removing DNA damage

**DOI:** 10.1038/s41467-017-02175-y

**Published:** 2018-01-26

**Authors:** Amy M. Whitaker, Tony S. Flynn, Bret D. Freudenthal

**Affiliations:** Department of Biochemistry and Molecular Biology, University of Kansas Medical Center, Kansas City, KS 66160 USA

## Abstract

Human apurinic/apyrimidinic (AP) endonuclease 1 (APE1) is an essential DNA repair enzyme which uses a single active site to process DNA damage via two distinct activities: (1) AP-endonuclease and (2) 3′ to 5′ exonuclease. The AP-endonuclease activity cleaves at AP-sites, while the exonuclease activity excises bulkier 3′ mismatches and DNA damage to generate clean DNA ends suitable for downstream repair. Molecular details of the exonuclease reaction and how one active site can accommodate various toxic DNA repair intermediates remains elusive despite being biologically important. Here, we report multiple high-resolution APE1–DNA structural snapshots revealing how APE1 removes 3′ mismatches and DNA damage by placing the 3′ group within the intra-helical DNA cavity via a non-base flipping mechanism. This process is facilitated by a DNA nick, instability of a mismatched/damaged base, and bending of the DNA. These results illustrate how APE1 cleanses DNA dirty-ends to generate suitable substrates for downstream repair enzymes.

## Abstract (web summary)

The essential DNA repair enzyme apurinic/apyrimidinic endonuclease 1 (APE1) has both endonuclease and exonuclease activities. Here, the authors present DNA bound human APE1 crystal structures which give insights into its exonuclease mechanism.

## Introduction

**Fig. 1:**
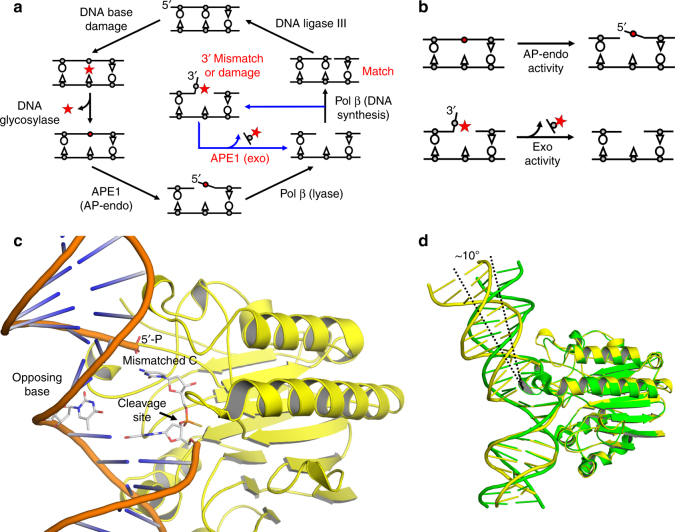
APE1 is multifunctional during BER. **a** Schematic of the BER pathway (black) showing the role of APE1 AP-endo activity and a proposed role for the APE1 exo activity (blue) in proofreading 3ʹ mismatches and removing 3ʹ end damage. DNA damage and mismatches are highlighted in red. **b** APE1 AP-endo and exo functions. **c** Overview of high-resolution APE1-DNA exo substrate complex. The site of cleavage is indicated by the arrow. The 3ʹ mismatched cytosine, its flanking base, and the opposing base are shown in stick format (gray carbons). **d** An overlay of the APE1 exo substrate complex in yellow with APE1 AP-endo substrate complex (PDB 5DFI, green) showing the difference in DNA bending

Nucleobase structures and base-pairing properties are altered by DNA damage, potentially leading to genomic mutations. Consequently, DNA damage is a major source of the mutation load that gives rise to numerous human maladies. To maintain genomic integrity, base excision repair (BER) is tasked with removing and replacing DNA lesions generated by oxidation and spontaneous base loss^1^. The BER pathway (Fig. 1a) is comprised of several factors; the scaffolding protein XRCC1, a damage-specific DNA glycosylase to remove the damaged base, apurinic/apyrimidinic endonuclease (APE1) to cleave the DNA backbone 5′ of the resulting apurinic/apyrimidinic (AP) site, DNA polymerase (pol) β to synthesize the DNA, and DNA ligase III to seal the repaired DNA. Gap filling synthesis by pol β requires a 3′-OH, necessitating removal of DNA damage at the 3′ end prior to repair. This cleaning of 3′ “dirty ends” can be performed by APE1^2–4^. Additionally, proper BER requires pol β to choose the correct nucleotide during DNA synthesis. Because pol β has only moderate fidelity compared to the replicative pols δ and ε, and lacks a proofreading domain, misinsertions during BER can occur and must be removed by a surrogate enzyme(s) in order to avoid genomic mutations^5,6^. APE1 has been proposed to serve this vital proofreading role during BER because of its established protein-protein interactions with pol β and ability to remove 3′ mismatches^2,7,8^. These additional activities of APE1 result in a modified version of the BER cycle in which APE1 can perform multiple functions (Fig. 1a, blue arrow).

Two predominant APE1 DNA cleavage reactions are (1) AP-endonuclease (AP-endo) and (2) exonuclease (exo) (Fig. 1b). Biochemical studies have shown that the AP-endo activity is more efficient in vitro, and the role of the exo activity has remained poorly understood in comparison^9^. Structural characterization of the APE1 AP-endo reaction demonstrated that the AP-site is flipped out of the DNA helical cavity and into the active site, allowing for phosphodiester bond cleavage to occur via a single metal ion and nucleophilic water^10,11^. APE1 exo activity removes 3′ end groups, including oxidatively damaged DNA bases, chain terminating nucleotide analog drugs, terminal blocking groups, and mismatched bases^2–4,12,13^. Despite the important biological implications of the exo activity, key mechanistic features of the exo reaction remain elusive. One major enigma is how the active site can form compactly around a baseless AP-site, but also accommodate the diversity of known exo substrates, including mismatched and modified bases^11^. To address this, we first utilized a mismatched nicked DNA substrate, representing a BER intermediate generated after incorrect nucleotide insertion by pol β (Fig. 1a), to structurally and kinetically characterize the APE1 exo mechanism. This study was expanded further to examine the cleansing of DNA dirty ends that arise during oxidative stress and would be blocks to subsequent replication and repair. These structures provide insight into how one active site can accommodate both AP-DNA and multiple exo substrates utilizing two different types of DNA backbone cleavage.

## Results

### APE1 bound to a mismatched DNA exonuclease substrate

We obtained a structure of APE1 engaged with a double stranded DNA substrate which contains a C/T mismatch at the 3′ end and a phosphate at the 5′ end of a nick. This substrate represents a BER intermediate in which pol β has misinserted a cytosine (C) across from a templating thymine (T) (Fig. 1a, blue arrow). A C/T mismatch was chosen because observations in our lab, as well as data published by others, indicate it as an optimal substrate for APE1 exo activity^14–17^. Initial substrate complex crystals were generated in a solution containing CaCl_2_, which inhibits catalysis^18,19^, and resulted in clear electron density for the phosphate and sugar moiety. However, relatively poor density was observed for the base of the mismatched C. To confirm we obtained an uncut substrate complex, we used otherwise identical DNA with a phosphorothioate (PS) modification 5′ to the mismatched C. This modification substitutes sulfur for a non-bridging oxygen, preventing incision^11,20,21^. The resulting substrate complex diffracted to 2.10 Å (Table 1). Of note, density for the mismatched C in both the PS and CaCl_2_ generated substrate complexes is similar, confirming that crystals grown in CaCl_2_ are substrate complexes.

**Table 1:** Data collection and refinement statistics

	3′ Mismatch	3′ Mismatch (MnCl_2_)	3′ Mismatch (product)	3′ Match	3′ PG	3′ Mismatch (F266A)
*Data collection*						
Space group	P2_1_	P2_1_	P2_1_	P_1_	P2_1_	P2_1_
Cell dimensions						
* a*, *b*, *c* (Å)	71.3, 63.8, 90.9	71.5, 64.4, 91.5	71.9, 63.5, 90.5	44.3, 61.9, 73.8	71.4,64.3,91.1	71.1, 65.5, 91.4
*α*, *β*, *γ* (°)	90, 109.7, 90	90, 109.9, 90	90, 109.4, 90	82.6, 76.9, 85.9	90, 110.1, 90	90, 110.1, 90
Resolution (Å)	25–2.10	25–2.20	25–2.3	25–2.6	25–2.3	25–2.0
*R*_meas_^a^(%)	10.6 (85.1)	9.4 (>100%)	12.4 (93.9)	14.6 (67.6)	10.5 (75.7)	8.4 (>100%)
*I*/σ*I*	13.7 (1.2)	24.4 (1.1)	15.3 (1.3)	13.8 (2.0)	15.6 (1.8)	18.4 (1.4)
*cc*1/2^b^	0.692	0.644	0.757	0.771	0.775	0.758
Completeness^a^ (%)	95.1 (87.3)	99.8 (98.6)	99.4 (99.4)	99.6 (98.5)	99.8 (99.8)	99.9 (99.9)
Redundancy^a^	4.0 (2.9)	11.2 (2.6)	4.2 (2.5)	4.0 (3.0)	4.5 (2.5)	5.0 (4.4)
*Refinement*						
Resolution (Å)	25–2.10	25–2.20	25–2.3	25–2.6	25–2.3	25–2.0
No. reflections	71,157	74,864	58,422	43,970	64,984	53,376
*R*_work_/*R*_free_	20.4/24.9	22.9/26.5	21.3/27.0	21.7/28.7	18.9/24.0	21.0/25.8
No. atoms						
Protein	4380	4371	4354	4306	4389	4381
DNA	955	917	834	834	871	955
Water	183	163	166	77	243	244
*B*-factors (Å^2^)						
Protein	46.5	49.1	44.9	43.1	33.0	36.4
DNA	78.1	80.1	63.3	54.7	55.5	71.0
3′ Base/PG DNA	52.4	62.2	N/A	37.1	35.9	47.9
Water	46.7	47.8	45.1	37.8	35.2	39.5
R.m.s deviations						
Bond length (Å)	0.009	0.011	0.010	0.012	0.008	0.011
Bond angles (°)	1.065	1.256	1.161	1.440	1.126	1.298
PDB ID	5WN4	5WN5	5WN1	5WN0	5WN2	5WN3

^a^Highest resolution shell is shown in parentheses.

^b^For highest resolution shell

**Fig. 2:**
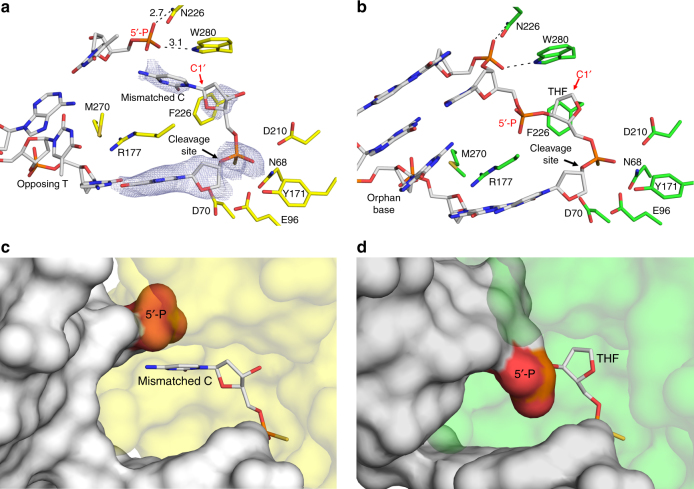
The APE1 exo substrate complex. **a**, **b** Active site of APE1 **a** exo and **b** endo (PDB 5DFI) substrate complexes with key residues indicated. The C1ʹ of the 3ʹ mismatched base and THF are indicated by red arrows. The cleavage sites are indicated by black arrows. 2*F*_o_–*F*_c_ map density (1*σ*) for the 3′ mismatched and flanking base is shown in light blue. **c**, **d** Surface representation of **c** exo and **d** endo active sites showing the varying bends of the DNA and displacement of the exo 5ʹ-P to vacate the active site for the 3ʹ mismatched base. The 5′-P is shown as surface in red. The protein side chains are shown in yellow (exo) or green (endo) and DNA residues in gray

**Fig. 3:**
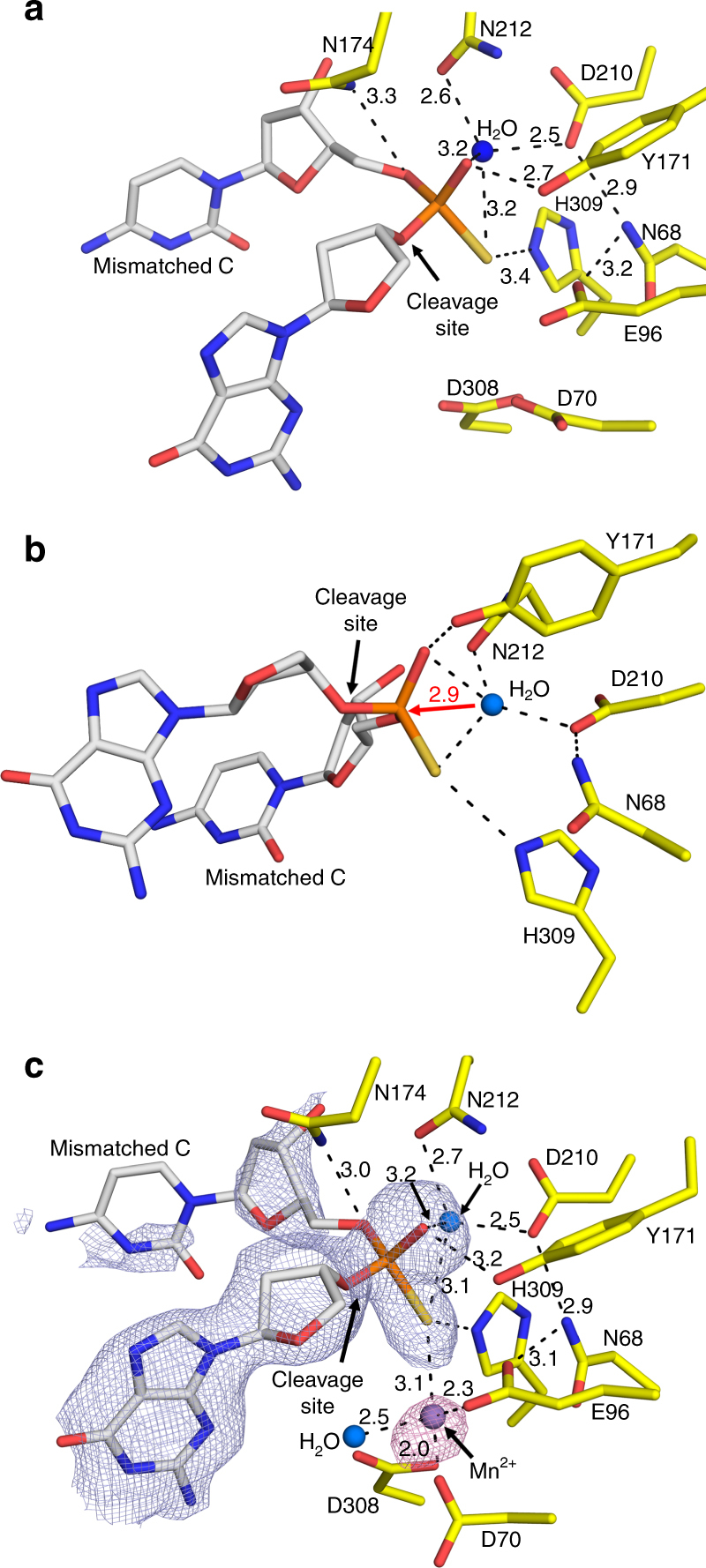
The APE1 exo substrate complex active site. **a** Focused view of the APE1 exo active site showing key residues, cleavage site, and nucleophilic water with distances (Å) indicated. **b** An alternative angle highlighting the nucleophilic water in position for attack of the phosphorus atom with the distance indicated. **c** The complete APE1 active site after soaking in MnCl_2_, with key residues indicated. 2*F*_o_–*F*_c_ map density (1*σ*) for mismatched C and the flanking base are shown in light blue. Anomalous map density (3*σ*) is shown for the MnCl_2_ in purple. The protein side chains are shown in yellow and DNA residues in gray. Water molecules and Mn^2+^ are shown as blue and purple spheres, respectively

The APE1 exo substrate structure revealed the mismatched C is in position for removal by incision of the 5′-phosphate backbone opposite an “opposing” T base and a bend in the DNA (Fig. 1c, d). In this conformation the mismatched C is positioned out of the protein active site into the open intra-helical cavity and the 3′ end of the DNA nick and phosphate backbone shift up and into the protein active site for cleavage. This is in stark contrast to the APE1 AP-endo cleavage mechanism which flips the AP-site into the protein active site, away from the DNA helix, to orient the phosphate backbone for cleavage. The active site for APE1 AP-endo and exo are shown in Fig. 2a, b with the C1′ position of the nucleoside sugar indicated for orientation. The C1′ position for the mismatched C in the APE1 exo structure is shifted ~24° and is 1.4 Å from its position in the APE1 AP-endo complex. The mismatched base does not make any apparent contacts with the protein active site, but appears to be sterically restricted into the intra-helical position by F266 and W280 (Fig. 2a). The opening up of the intra-helical cavity in the APE1 exo substrate structure occurs via several perturbations, including a ~10° sharper bend in the conformation of the DNA (Fig. 1d), destabilization of residue R177 (*B*-factor 66.9 Å^2^ compared to 26.7 Å^2^), and M270 acting as a wedge to coordinate the opposing T away from the mismatched C (Fig. 2a). The 5′-phosphate is coordinated by W280 and N226, as well as the oxygen (O2) of the mismatched C (Fig. 2a). Both the nick in the DNA and the shifted positon of the phosphate at the 5′ end allows for the greater bend in the DNA and space for the mismatched base while keeping the phosphate backbone in positon for catalysis (Fig. 2c, d). The phosphate backbone oxygen (O5′) is coordinated to N174 (ND2) and N212 (OD1) (Fig. 3a). The 3′-OH of the sugar is also coordinated to N174, while Y171 and H309/E96 coordinate the non-bridging oxygen and sulfur, respectively. Importantly, a well-ordered water molecule is in position to act as the nucleophile (Fig. 3a, b). This water is coordinated by the non-bridging oxygen and sulfur atoms of the backbone phosphate, positioning the water oxygen atom 2.9 Å from the phosphorus atom (Fig. 3b). The oxygen atoms of N212 and D210 come within hydrogen bonding distance of the nucleophilic water molecule at 2.6 and 2.5 Å, respectively. Also observed is a hydrogen bonding network between E96, N68, D210, and N212 that likely alters the p*K*_a_ of D210, thus facilitating the deprotonation of the water and subsequent nucleophilic attack at the phosphate backbone during cleavage. These contacts are consistent with a water mediated nucleophilic attack mechanism.

The APE1 exo reaction has been reported to be metal dependent, however our structures reported above lack a clearly identifiable metal ion, even in the presence of CaCl_2_. Identifying the number and binding site of metal ions for APE1 cleavage has been historically challenging^11,22,23^. To determine the metal binding site(s) used for the exo reaction, we briefly soaked PS substrate crystals of the APE1 exo complex in a cryosolution containing MnCl_2_ and determined a 2.20 Å substrate complex (Table 1). Using the anomalous signal from Mn^2+^, we determined the position of a single, catalytic metal coordinated by D308, D70, E96, and the phosphate backbone of the mismatched C (Fig. 3c). The location of the catalytic metal is identical to that in the APE1 AP-endo structure and is adjacent to the site of cleavage, poised to facilitate catalysis and stabilize reaction intermediates. All other contacts are identical to those observed in the metal free APE1 exo substrate complex, described above, including the nucleophilic water bound within the active site (Fig. 3).

### APE1 exonuclease product and matched DNA substrate complexes

**Fig. 4:**
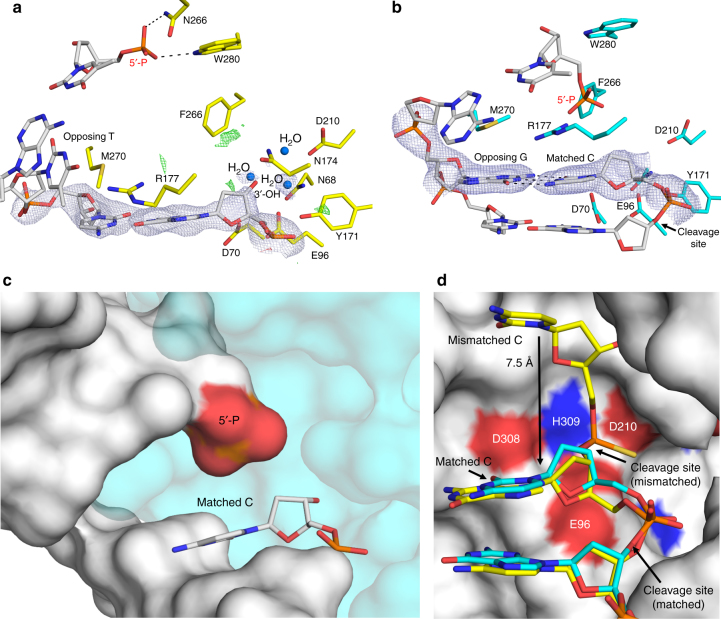
Structures of APE1 bound to a 3ʹ mismatched base exo product and a matched DNA substrate. **a** APE1 exo C/T mismatched product complex. 2*F*_o_–*F*_c_ map density (1*σ*) is shown in light blue. Positive (green) and negative (red) omit map densities are shown 4 Å from the 3′-OH at +3 and −3*σ*, respectively. **b** APE1 exo C/G match substrate complex. The base pairing interaction for the C/G base pair is shown as dashes. 2*F*_o_–*F*_c_ map density (1*σ*) is shown in light blue. **c** APE1 exo C/G match substrate complex in surface representation. The 5′-P is highlighted in red. **d** Overlay of the APE1 substrate complex with a C/T mismatch and C/G match at the 3ʹ end of a nick with DNA in yellow and cyan, respectively. Protein is from the 3′ mismatched structure and is shown in surface representation. The shift in location of the cleavage sites between the mismatched and matched structures is indicated by an arrow. For each structure, waters are shown as blue spheres and key residues, the site of cleavage, and distances (Å) are indicated

The APE1 exo activity can proofread pol β misinsertions to regenerate a 1-nt gapped DNA product, providing a second chance for correct (matched) pol β gap filling DNA synthesis (Fig. 1a)^7,8^. To obtain an APE1 exo product structure, we first grew an exo substrate complex crystal in the presence of CaCl_2_ and subsequently transferred it to a cryosolution containing MgCl_2_ to initiate catalysis. This approach relies on APE1 to perform backbone cleavage, excising the base, in crystallo. The resulting crystal was flash frozen after soaking for 2.5 h and diffracted to 2.3 Å (Table 1). This structure shows APE1 in complex with its single nucleotide gapped DNA product, revealing the mismatched base has been removed (Fig. 4a). Importantly, because this complex was generated in crystallo with an APE1 substrate crystal, it verifies that our substrate structures represent APE1 in the catalytically competent conformation for exo activity. The newly generated 3′-OH is stabilized by a hydrogen bonding network involving N212, D210, Y171, N174, H309, E96, N68, and three water molecules (Fig. 4a). The phosphate at the 5′ end of the nick is coordinated by N222, W280, and two waters. The product complex lacks a clearly identifiable metal ion within the active site, even with high concentrations of MgCl_2_ present in the soak. This is consistent with previous structural snapshots indicating high disorder for Mg^2+^ within the active site^11,22,23^.

APE1 exo activity removes 3′ mismatched nucleotides at least 50 times more efficiently than matched nucleotides^2^. Steady-state kinetic assays determined the apparent binding constants (*K*_M_) for APE1 to be similar for mismatched and matched DNA substrates, with the increased efficiency of mismatch removal attributed to a higher rate of catalysis^12^. To gain insight into substrate specificity of APE1 exo activity between 3′ mismatched and matched DNA, we determined the structure of APE1 bound to a double stranded DNA substrate containing a C/G match at the 3′ end and a phosphate at the 5′ end of a nick (2.6 Å, Table 1). In this structure, Watson–Crick base pairing is retained between C and G at the 3′ end of the nick (Fig. 4b), unlike what was observed in the mismatched C/T structure. The stable C/G base pairing prevents the phosphate backbone from entering the proper registry for cleavage by the APE1 active site (Fig. 4c). An overlay of the C/G and C/T APE1 exo structures, Fig. 4d, reveals the matched C is shifted 7.5 Å downstream and away from the key catalytic residues for exo cleavage. APE1 likely removes a matched base using the same mechanism as a mismatched base, just with a lower efficiency due to the relative lack of flexibility at the 3′ end of the nick, as demonstrated by stable base pairing and low *B*-factor (36.21 Å^2^) for C in our C/G structure. This result is in agreement with previous studies showing that the efficiency of the APE1 exo reaction is directly correlated to the thermal stability of its duplex DNA substrate as dictated by the 3′-terminal base pair (with a match being the most stable and having the weakest activity)^17^.

### APE1 poised to remove oxidative DNA damage at the 3′ end

**Fig. 5:**
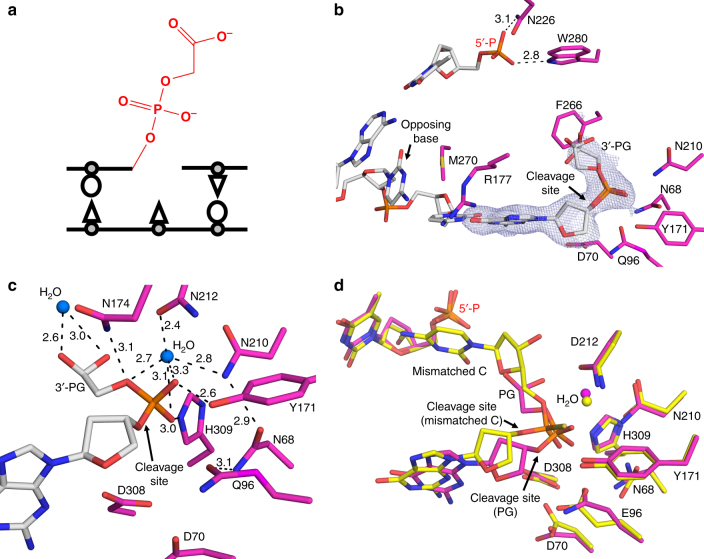
Structure of APE1 poised to remove a 3ʹ-PG. **a** Schematic showing the structure of PG at the 3ʹ end of a DNA nick at neutral pH. **b** APE1 exo 3ʹ-PG substrate complex. 2*F*_o_–*F*_c_ map density (1*σ*) is shown in light blue. The two 3ʹ-PG conformations are shown. **c** A close-up of the APE1 3ʹ-PG substrate complex active site. The protein side chains are shown in magenta and DNA residues in gray. Waters are shown as blue spheres and key residues, the site of cleavage, and distances (Å) are indicated. **d** An overlay of the 3ʹ-PG (magenta) substrate complex with the 3ʹ mismatched (yellow) substrate complex

The APE1 exo activity is also involved in the cleaning of DNA “dirty ends”, including DNA damage generated by reactive oxygen species (ROS) during oxidative stress. ROS-induced DNA damage is both a major driver of human disease and a product of environmental exposure, radiation, and chemotherapeutic cancer treatments^24,25^. One common 3′ damaged terminal end generated by ROS is phosphoglycolate (3′-PG, Fig. 5a). This stable end product blocks replication and repair, and it must be removed prior to further synthesis by a DNA polymerase. While multiple enzymes are capable of removing 3′-PG ends, APE1 has been shown to be the major enzyme responsible for this activity^26–29^. In the context of BER, APE1 is capable of processing PG at the 3′ end of a nick to produce a 1-nt gapped DNA substrate with a 3′-OH suitable for pol β gap filling DNA synthesis. To gain insight into how APE1 processes damaged DNA ends, we determined the crystal structure of APE1 bound to a double stranded, nicked DNA substrate containing a PG at the 3′ end and a phosphate at the 5′ end of the nick (2.3 Å, Table 1). In this structure, APE1 binds 3′-PG in two alternate conformations, both situating the cleavage site phosphate group in a similar position to what we observed for a 3′ mismatched base (Fig. 5b). However, in the absence of an intact nucleobase at the 3′ end, several waters are observed in the more spacious active site poised to coordinate both ends of the 3′-PG/nicked DNA substrate (Fig. 5c). One water is in position to coordinate both of the PG carbonyl oxygens, at 2.6 and 3.0 Å. Both the cleavage site phosphate and the nucleophilic water are positioned and coordinated almost identically as shown in the mismatched exo substrate structures (Fig. 5d). This is consistent with a universal APE1 3′ to 5′ exo cleavage mechanism among the diverse substrates of PG and a mismatched base.

### Further characterization of the APE1 exonuclease reaction

**Fig. 6:**
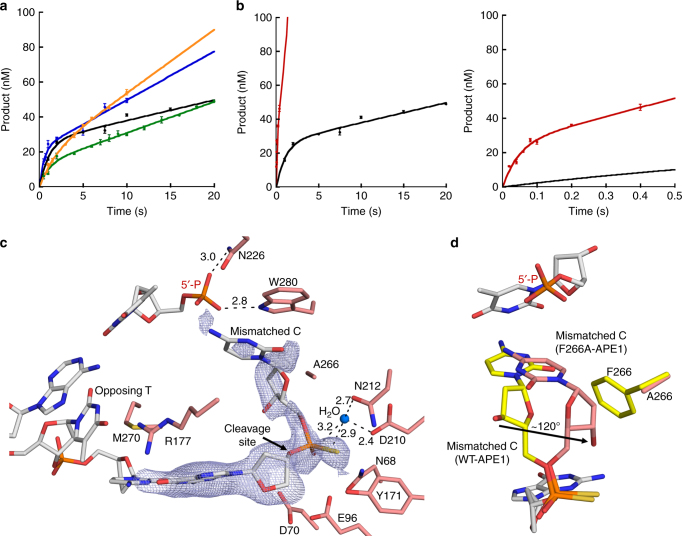
Kinetic and mutant characterization of the APE1 exo reaction. **a** Pre-steady-state kinetic time courses for the APE1 exo reaction are shown in the left panel. Curves shown are for WT APE1 (black), M270A (blue), R177A (green), and W280A (orange). **b** Time course of product formation for F266A (red) in comparison to WT (black). The early time points are shown in the right panel. For all time courses, the line represents the best fit to an equation with a rising exponential and linear term. Kinetic parameters are shown in Table 2. All error bars represent the standard error of the mean from three replications of the experiment. **c** Structure of the F266A mutant exo substrate complex active site. 2*F*_o_–*F*_c_ map density (1*σ*) is shown in light blue. **d** An overlay of the F266A exo substrate structure (salmon) with the WT substrate structure (light gray DNA, yellow protein). An arrow indicates the rotation of the 3ʹ mismatch sugar moiety. Key residues, the site of cleavage, and distances (Å) are indicated

Our structures demonstrate important distinctions between the incision reactions of APE1 during AP-endo and exo functions, suggesting the recognition and repair chemistry of APE1 may be different for these two types of substrates. To probe the APE1 exo mechanism, we conducted pre-steady-state kinetic measurements of APE1 removing a mismatched C, opposite T, at the 3′ end of a centrally located nick in duplex DNA. We utilized a C/T mismatched substrate for characterization of the APE1 exo mechanism because this experimental approach (C nucleobase removal) allows for clearer separation between substrate and product bands on a gel compared to removal of the smaller 3′-PG end damage. Also, the PG and the mismatched C are in an identical location for the APE1 exo activity (Fig. 5d). Kinetic time courses for wild-type APE1 with an exo substrate are compared to those previously obtained with a double stranded DNA AP-endo substrate containing a centrally located AP-site analog (tetrahydrofuran, THF)^11^. We obtained biphasic time courses of product formation for the exo substrate, demonstrating that catalysis during the first enzymatic turnover (burst phase) is more rapid than in the subsequent steady-state phase where a step after chemistry (likely product release) is rate limiting (Fig. 6a and Table 2)^30^. For WT-APE1 with a C/T mismatched exo substrate, the observed rate constant of the burst phase was 0.9 ± 0.2 s^−1^. With this analysis, the burst amplitude represents the apparent active enzyme concentration (26 ± 2 nM) so that the steady-state (*k*_ss_ = *v*_ss_/[APE1_active_]) was 0.045 ± 0.008 s^−1^. Comparing these rates obtained for WT-APE1 with the exo substrate to those with an AP-endo substrate demonstrate that the burst rate and steady-state rates for the APE1-endo reaction are only ~40 and ~18-fold faster than those for the APE1 exo reaction, respectively.

**Table 2:** Pre-steady-state kinetic parameters of the WT and mutant APE1 proteins

APE1 Variant	[APE1]_active_ (nM)	*k*_obs_ (s^−1^)	*k*_ss_ (s^−1^)
*3′ mismatch (exo)*			
WT	26 ± 2	0.9 ± 0.2	0.045 ± 0.008
F266A	25 ± 5	21 ± 7	2.1 ± 0.7
M270A	22 ± 4	1.9 ± 0.9	0.13 ± 0.03
R177A	13 ± 1	1.0 ± 0.2	0.14 ± 0.01
W280A	17 ± 4	0.5 ± 0.2	0.21 ± 0.05
*THF (endo)*			
WT^a^	11	36 ± 6	0.8 ± 0.1

^a^ Freudenthal, B. D. et al.^11^

To probe the role of several active site residues (M270, R177, W280, and F266), we performed pre-steady-state kinetic measurements on the same nicked 3′ C/T mismatched substrate used above with APE1 variant enzymes (Fig. 6a, b and Table 2). Our APE1 exo structures show M270 wedged between the mismatched bases, leading us to propose that it may play a role destabilizing the base pairing interactions. However, when this residue was mutated to an alanine both the observed burst rate and the steady-state rate were moderately increased compared to wild-type APE1, at 1.9 ± 0.9 s^−1^ and 0.13 ± 0.03 s^−1^ respectively, leaving the role of M270 in the exonuclease reaction ambiguous. R177, which has been proposed to facilitate slow product release in the APE1 AP-endo reaction by interacting with the orphan base, was displaced and/or disordered in our exo structures. In agreement with R177 having a neutral role in the exo reaction, kinetic characterization of the R177A APE1 mutant produced an observed burst rate similar to wild type of 1.0 ± 0.2 s^−1^ and a slightly increased *k*_ss_ of 0.14 ± 0.01 s^−1^. Our structures indicate that the side chains of both W280 and F266 reduce the size of the APE1 active site, and previous steady-state kinetic analyses indicate W280A and F266A APE1 mutants increase the exo catalytic rate^31^. In agreement, W280A APE1 showed a ~fivefold increase in the steady-state rate (*k*_ss_ = 0.21 ± 0.05 s^−1^) compared to wild type. However, there was also a ~twofold decrease in the observed burst rate (*k*_obs_ = 0.5 ± 0.2 s^−1^). This indicates that W280 may play a role in the slow product release step of the exo reaction, while also having a modest impact on the incision step.

The F266A APE1 mutation had the most dramatic effect on exo activity, with a ~20-fold increase in the observed burst rate and a ~50-fold increase in the steady-state rate at 21 ± 7 s^−1^ and 2.1 ± 0.7 s^−1^, respectively (Fig. 6b and Table 2). Notably, this burst rate is close to what we observed for the APE1 AP-endo reaction (<2-fold difference) and the steady-state rate exceeds that observed for the AP-endo reaction by ~threefold (Table 2). Interestingly, F266A does not have the same effect on the AP-endo reaction^32,33^. To further elucidate the catalytic role of F266 in the exo reaction, we obtained a 2.0 Å substrate complex structure of F266A APE1 bound to DNA containing a C/T mismatch at the 3′ end of a nick and a PS linkage modification 5′ to the C (Table 1). The structure revealed that mutating F266 to an alanine expands the size of the APE1 active site, allowing the mismatched C to occupy an alternate conformation (Fig. 6c). When the mismatched C and F266 in the WT-APE1 structure are overlaid with those in the F266A APE1 structure, it is revealed that the mismatched C has rotated by ~120° in the mutant structure into a part of the active site that is normally occupied by the F266 side chain (Fig. 6d).

## Discussion

The biological significance of APE1 is highlighted by embryonic lethality in mice where its expression is knocked out, expression of inactive variants enhancing cellular sensitivity to DNA damaging therapeutics, and polymorphisms associated with an increased cancer risk^34–36^. How a single APE1 active site can have various biological activities on an array of nucleic acid substrates has been of interest to researchers for decades^2,8,11,26,37–39^. A key aspect of the APE1 AP-endo reaction is the flipping of the AP-site into the active site during cleavage. Our structures demonstrate APE1 uses the same active site to exonucleically remove 3′ mismatches and DNA damage by a non-base flipping mechanism. Instead, key mechanistic features include the 3′ end of a DNA nick sliding into the APE1 active site where it is stabilized by protein contacts, the instability of the 3′ end resulting from the mismatch/damage, and DNA bending^9,14–16^. Also, several residues are positioned to interact with the 5′ phosphate, providing an explanation for differences in incision efficiency based on the nature of the 5′ terminal group^9,17^. These DNA-centric mechanistic features are present in cases of other 3′ bulky end damages and nucleotide analogs, indicating the mechanism could be universal for APE1 exo function^40^. Importantly, our structures show that the APE1 active site is quite rigid and incision differences between substrates likely results from altered DNA conformations, as opposed to APE1 conformational changes^12^.

The relatively weak exo activity of APE1 in comparison to its AP-endo function has been primarily attributed to a hindered catalytic reaction (*k*_cat_)^9,41^. Our kinetics show that rates for both the chemistry step and the product release step of the exo reaction on a mismatch are reduced in comparison to the AP-endo reaction by only ~40-fold and ~18-fold, respectively. This reduction in rate is recovered by the single F266A mutation, which increases space in the APE1 active site and allows the mismatched base to adopt an alternative conformation (Fig. 6, Table 2). A restrictive active site and larger exo substrate may be responsible for the reduced incision rate for the APE1 exo reaction. The hydrophobic pocket, comprised of F266 and W280, is not well conserved and appears to play a role in substrate specificity between ExoIII family members^31^. To this point, *Escherichia coli* ExoIII possesses a hydrophobic pocket composed of only one aromatic residue, and exhibits a relatively strong 3′ to 5′ exonuclease activity. Similarly, members of the APE2 subfamily completely lack this hydrophobic pocket and have a strong preference for exo substrates compared to AP-endo substrates^31,42–44^.

At estimated in vivo APE1 protein concentrations, the exo activity of APE1 is rather robust in vitro, and thus is presumably regulated by additional cellular factors and/or is directed to its preferred substrates^9,45^. Our structures demonstrate that flexibility at the 3′ end (from a mismatch or DNA damage) confers at least some specificity, supporting a biological role for APE1 exo activity in proofreading misinsertions and removal of 3′ dirty ends during BER to generate suitable substrates for downstream repair enzymes. The BER ligation reaction has a demonstrated dependence on the presence of APE1 in reconstituted BER systems^2^. Moreover, the lower efficiency of DNA ligase in rejoining mispaired DNA creates a window of time for APE1 to remove the mismatched nucleotide. Although other 3′ to 5′ DNA exonucleases have been proposed to potentially provide a proofreading function for DNA pol β, APE1 is conceptually more suitable for this task as its physical interaction with DNA pol β has already been shown^46–48^.

It is of interest to consider whether or not the APE1 exo mechanism could be used to perform other DNA cleavage reactions attributed to APE1. For example, APE1 performs backbone cleavage during the nucleotide incision repair (NIR) pathway^49,50^. APE1 NIR activity consists of incision at the 5′ side of a damaged base in a DNA glycosylase-independent manner, followed by the subsequent repair through downstream factors^14,51–53^. Interestingly, the optimal conditions for NIR activity are very similar to those required for the APE1 exo activity, and differ from those for the AP-endo activity^49^. Furthermore, a role is emerging for APE1 endoribonuclease cleavage in the post-translational regulation of RNAs, such as c-myc^37,54–56^. As these substrates would require considerable active site real-estate, APE1 likely uses an intra-helical exo-like mechanism during NIR and RNA cleavage. The structures presented here provide valuable insight into how APE1 utilizes the same active site to cleave small AP-sites and bulkier substrates, including the proofreading of mismatches and the cleaning-up of DNA damage that blocks DNA replication and repair.

## Methods

### DNA sequences

To generate the 21-mer nicked duplex for crystallization, the following DNA sequences were used: matched opposing strand, 5′-GGA-TCC-GTC-GAG-CGC-ATC-AGC-3′; mismatched opposing strand, 5′-GGA-TCC-GTC-GAT-CGC-ATC-AGC-3′; upstream strand with 3′ cytosine, 5′-GCT-GAT-GCG-C-3′; upsteam stand with 3′-PG, 5′-GCT-GAT-GCG-(PG)-3′; downstream strand with a 5′-phosphate, 5′-TCG-ACG-GAT-CC-3′. To generate the 30-mer for the kinetic studies the following DNA sequences were used: matched opposing strand, 5′- ATG-CGG-ATC-CGT-CGA-GCG-CAT-CAG-CGA-ACG-3′; mismatched opposing strand, 5′-ATG-CGG-ATC-CGT-CGA-TCG-CAT-CAG-CGA-ACG-3′; upstream strand with a fluorescein (indicated by astrick), 5′-*CGT-TCG-CTG-ATG-CGC-3′; downstream strand with a 5′-phosphate, 5′-TCG-ACG-GAT-CCG-CAT-3′. All sequences were purchased from IDT, except for the PG containing sequence which was purchased from Eurogentec. The oligonucleotides were separated from other DNA species by electrophoresis on a 16% polyacrylamide gel containing 7 M urea in TBE buffer. Purified DNA substrates were annealed in buffer containing 50 mM tris and 50 mM KCl, and the concentration was determined by absorbance at 260 nm.

### Protein expression and purification

Human wild-type and truncated APE1 (lacking 43 N-terminal amino acids, ΔAPE1) were expressed from pet28a codon optimized clones purchased from GeneScript. All mutagenesis was carried out in either the full-length or truncated clones using QuikChange II site-directed mutagenesis (Agilent) and the primer sequences are shown in Supplementary Table 1. APE1 was expressed in One Shot BL21(DE3)plysS *E*. *coli* cells (Invitrogen), grown at 37 °C until induced at OD = 0.6, and then grown overnight at 20 °C. After harvesting, cells were lysed at 4 °C by sonication in 50 mM HEPES, pH 7.4, 50 mM NaCl, and a protease inhibitor cocktail. The lysate was pelleted at 24,242 ×* g* for 1 h. The resulting supernatant was passed over a HiTrap Heparin HP (GE Health Sciences) equilibrated with lysate buffer. APE1 was eluting from the heparin column with a linear gradient of NaCl up to 1 M. APE1 eluting at high salt was buffer exchanged into 50 mM NaCl and loaded onto a POROS HS cation exchange column (GE Health Sciences) and eluted with a linear gradient of NaCl up to 1 M. Purified APE1 was subsequently loaded onto a HiPrep 16/60 Sephacryl S-200 HR (GE Health Sciences). The resulting pure fractions were concentrated and stored at −80 °C. Final concentrations was determined by NanoDrop One UV–Vis Spectrophotometer (Thermo Scientific).

### Crystallization and structure determination

DNA substrates for APE1–DNA complex crystals were made by annealing 2 mM of three oligonucleotides in a 1:1:1 ratio to form a 21-mer duplex with a central nick using a PCR thermocycler by heating for 10 min at 90 °C and cooling to 4 °C (1 °C min^−1^). The annealed DNA was mixed with truncated C138A-APE1 to achieve a final concentration of 0.56 mM DNA and 10–12 mg ml^−1^ C138A-APE1. The single amino acid C138A mutation and truncation of the N-terminal 40 amino acids aid in crystallization^23^. APE1-DNA complexes were crystalized by vapor diffusion. The reservoir solution for crystal formation was 7–14% PEG 20 K, 100 mM sodium citrate, pH 5.0, 15% glycerol, and 5 mM CaCl_2_. Crystals grew within a week at 20 °C. APE1-DNA crystals were transferred to a cryosolution containing the mother liquor with 20% ethylene glycol. Data were collected at 100 K on a Rigaku MicroMax-007 HF rotating anode diffractometer equipped with a Dectris Pilatus3R 200K-A detector system at a wavelength of 1.54 Å. This allowed for anomalous data detection after phasing by molecular replacement with high redundancy. Data were processed and scaled with the HKL3000R software package^57^. Initial models were determined by molecular replacement with a modified version of a previously determined APE1–DNA complex (PDB 5DFF) as a reference. Refinement was carried out with PHENIX and model building with Coot^58,59^. The PS linkage containing substrates are present in two isomers, *S*_p_ and *R*_p_. In our crystal structures, we observed both isomers in the active site with equal occupancy (Supplementary Fig. 1). The figures were prepared with PyMOL (Schrödinger LLC), and for simplicity only one conformation is shown for residues with alternate conformations unless otherwise noted.

### Kinetic characterization

A rapid quench-flow instrument (KinTek RQF-3) was used for enzyme activity measurements. DNA substrates used to measure APE1 incision activity (37 °C) contained a 5′,6-carboxyfluorescein (6-FAM) label and were designed with a centrally located nick flanked by a 3′ mismatched cytosine and a 5′-phosphate. The reaction buffer was 50 mM HEPES, pH 7.4, 100 mM KCl, 3 mM MgCl_2_, and 0.1 mg ml^−1^ BSA. Final concentrations were 100 nM DNA substrate and 30 nM APE1 after mixing. At indicated time intervals, aliquots were quenched by mixing with 100 mM EDTA. An equal volume of DNA gel loading buffer (100 mM EDTA, 80% deionized formamide, 0.25 mg ml^−1^ bromophenol blue and 0.25 mg ml^−1^ xylene cyanol) was added to the quenched reaction mixture. After incubation at 95 °C for 5 min, the reaction products were separated by 16% denaturing polyacrylamide gel. All time points are the mean of three independent experiments. A GE Typhoon FLA 9500 imager in fluorescence mode was used for gel scanning and imaging, and the data were analyzed with Image J software^60^. The biphasic time courses were fit to the equation: Product = *A*(1−*e*^−kobst^) + *v*_ss_*t*, where *A* represents the amplitude of the rising exponential and *k*_obs_ the first order rate constant. The steady-state rate constant (*k*_ss_) is the steady-state velocity (*v*_ss_) A^−1^, where *A* represents the fraction of actively bound enzyme.

### Data availability

Coordinates and structure factors have been deposited in the Protein Data Bank under accession codes 5WN4, 5WN5, 5WN1, 5WN0, 5WN2, and 5WN3. Other data are available from the corresponding author upon reasonable request.

## Electronic supplementary material

Supplementary Information
