# Supplementary material for: Molecular snapshots of APE1 proofreading mismatches and removing DNA damage
Source: Nat Commun. 2018 Jan 26;9:399. doi: 10.1038/s41467-017-02175-y (PMC5785985; doi:10.1038/s41467-017-02175-y)
Supplement: Supplementary file 1 — Supplementary Information [file 41467_2017_2175_MOESM1_ESM.pdf]

Supplemental Table 1 Mutagenesis Primer Sequences

APE1 Variant	Primer 1	Primer 2
F266A	5'-caccccgtacgcgtataccgcttggacctatatgatgaac-3'	5'-gttcatcatataggtccaagcggtatacgcgtacggggtg-3'
M270A	5'-tgctacgcgcgttcatcgcataggtccaaaaggtatacgc-3'	5'-gcgtataccttttggacctatgcgatgaacgcgcgtagca-3'
R177A	5'-accaggccagcacccgcgttcggcacat-3'	5'-atgtgccgaacgcgggtgctggcctggt-3'
W280A	5'-gcgcgtagcaaaaacgttggtgcgcgtctggact-3'	5'-agtccagacgcgcaccaacgtttttgctacgcgc-3'

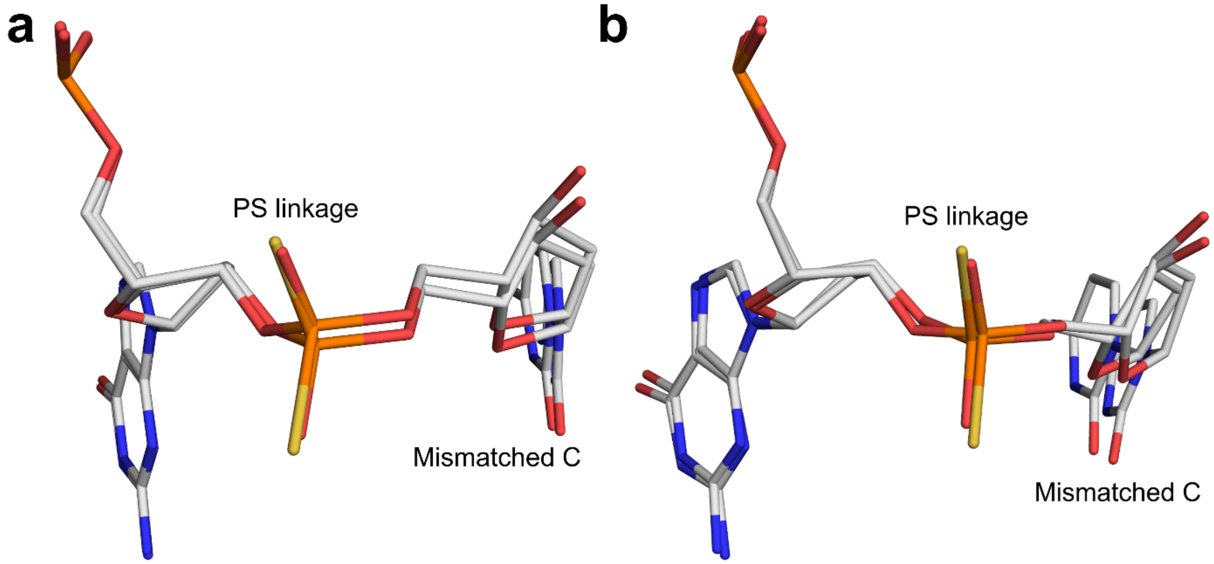

Supplementary Figure 1 The phosphorothioate stereoisomer species. (**a**) The mismatched C and two 5'-flanking phosphorothioate (PS) linkage isomers are shown in stick format. (**b**) The metal bound structure displays the same two conformers.
